# Mindfulness-based relapse prevention targeting psychological craving and trait mindfulness in young Chinese women with methamphetamine dependence: a randomized controlled trial

**DOI:** 10.3389/fpsyt.2024.1339517

**Published:** 2024-10-01

**Authors:** Xuan Liu, Yidan Zhang, Hongxin Cheng, Honglin Dong, Yuting You, Yuxi Wu, Chunli Yang, Lushi Jing

**Affiliations:** ^1^ School of Psychology, Chengdu Medical College, Chengdu, China; ^2^ Sichuan Women’s Compulsory Isolation Drug Treatment Center, Deyang, Sichuan, China

**Keywords:** MA-dependent, female, young adult, psychological craving, MBRP

## Abstract

**Methods:**

We recruited 58 MA-dependent young adult females from a compulsory isolation drug rehabilitation center in Sichuan Province and randomly divided them into an MBRP group (n = 29) and a control group (n = 29) according to their degree of psychological craving. The MBRP group received 2 hours of MBRP training twice a week for 4 weeks, alongside routine treatment at the drug rehabilitation center. Meanwhile, the control group solely received routine treatment at the drug rehabilitation center without any additional interventions. The assessment was conducted before and immediately after the intervention, with the Compulsive Drug Use Scale (OCDUS) used to assess craving and the Five-Factor Mindfulness Scale (FFMQ) used to assess trait mindfulness. Also, a “mental feedback monitoring balance” instrument was used to assess concentration and relaxation during some training sessions. This randomized trial was conducted to evaluate the effectiveness of decreasing psychological craving and increasing trait mindfulness.

**Results:**

At baseline, there were no significant differences in total or dimension scores for FFMQ or OCDUS between the two groups (all *P* > 0.05). After the intervention, the repeated measures ANOVA showed a significant time main effect on changes in observing, non-judging, and non-reacting scores (all *P* < 0.05), and a significant interaction effect between time and group on both FFMQ total score and OCDUS score (*P* < 0.01 or *P* < 0.05). Mental feedback monitoring indicated significant improvement in concentration and relaxation after breath meditation exercises (*P* < 0.05 or *P* < 0.001). Additionally, the MBRP group showed improved relaxation during the body scan exercise (*P* < 0.01).

**Conclusion:**

MBRP training can improve the trait mindfulness of MA addicts and reduce psychological cravings effectively.

## 1 Introduction

Methamphetamine (MA) is a psychotropic substance controlled under Chinese regulations, and the 2023 World Drug Report indicates that about 36 million people use amphetamine-type drugs, representing 0.7% of the global population (1). Unlike other types of substance abuse, over 45% of those who have used MA are female. However, women account for only 27% of those who receive drug treatment. Accumulating evidence suggests that female stimulant drug users may be more vulnerable to the development of problematic drug use patterns as compared to men, but many previous studies focused on young male MA adults (2). In addition, women seem to be more inclined to MA and less likely to use other alternative medications when unable to obtain MA. Young adulthood is a transitional phase from adolescence to full-fledged adulthood. The prevalence of alcohol and drug use peaks during young adulthood, potentially impeding critical developmental tasks and setting the stage for chronic substance misuse and associated social, educational, and health-related outcomes (3), so they warrant particular attention. Therefore, in this study, we choose MA-dependent young female adults so we can better understand the therapeutic effect of MBRP on MA users.

Regarding MA use disorder (MUD), craving is one of the core characteristics and a key factor in maintaining addictive behavior (4). Psychological craving (drug craving) can be defined as an intense, conscious desire for drugs (5). Several studies have shown that increased psychological craving significantly predicts substance use relapse (6), so reducing psychological craving may be an effective way to reduce the incidence of relapse among MA-dependent female youths. In China, drug abuse is illegal, and people who find it difficult to abstain from drug use or relapse during community detoxification face 2 years of compulsory isolation. In general, drug addicts can achieve complete physical detoxification through mandatory detoxification in drug rehabilitation centers, but it is difficult to get rid of the “mental addiction”, and more than 90% of drug addicts relapse after leaving a rehabilitation center (7). For this reason, many methods have been introduced to reduce psychological craving, such as art therapy, transcranial direct stimulation (tDCS), and motivational interviewing (MI), all of which have a certain degree of clinical efficacy. However, due to the need for long-term professional treatment or expensive equipment, these methods have some problems in the large-scale promotion of treatment (8). The elaborated intrusion (EI) theory of desire emphasizes the role of cognitive processes in experiencing and sustaining craving episodes. It holds that the initial source of desire is a learned association between a particular internal or external cue and a particular behavior. These, along with associated physiological responses, can lead to intrusive thoughts. When these thoughts arise and trigger a strong emotional response or sense of deficit, they can lead to cognitive elaboration (9). That is, to seek out or manipulate relevant information from memory to construct a vivid sensory image of the object of desire and related, resulting in a strong craving. Therefore, according to this theoretical model, anything that prevents or interrupts this fine process helps to prevent or limit the duration of craving episodes. In recent years, mindfulness has been introduced into the field of addiction treatment (10). Mindfulness-based relapse prevention (MBRP) combines meditation with traditional relapse prevention (RP) for the treatment of multiple substance addictions, such as alcohol and nicotine. MBRP improves awareness and acceptance of personal experiences through mindfulness meditation practices that allow a person to experience that his or her current physical and emotional state is transitory. At the same time, MBRP encourages practitioners to focus on the present moment, and to attend to thoughts and feelings with acceptance, openness, and non-judgmental attitudes, thus reducing “automatic” reactions and allowing addicts to break free from deep-seated beliefs and behaviors associated with drugs and drug use behavior (11), thus interrupting the cognitive fine-grained process of the desire invasion theory, and giving the addict the opportunity to break away from the deep-rooted beliefs (brief) and behavioral habits associated with the drug. Several studies have demonstrated MBRP training can improve trait mindfulness (or disposition mindfulness). Trait mindfulness is a naturally occurring mental quality that varies in different individuals. It refers to a present-oriented, nonjudgmental awareness of cognitions, emotion, perception, and habitual behavior in daily life, this dispositionality may strengthened through participation in MBRP training and other types of interventions (12). After undergoing MBRP training, substance addicts experience significant reductions in their cravings and relapse rates (13, 14). However, few studies on MBRP have been conducted on MA-dependent young female Chinese adults in compulsory drug treatment facilities, and objective measures of whether trait mindfulness improve treatment are lacking.

The study aimed to investigate whether mindfulness-based relapse prevention (MBRP) can help MA-dependent female youths reduce their psychological cravings and increase their trait mindfulness, which was objectively measured using a “mental feedback monitoring balance” instrument during the training process. The intervention will help MA-dependent female youths learn to be aware of their feelings and better regulate their behaviors, thus decreasing cravings and reducing the incidence of relapse.

## 2 Materials and methods

### 2.1 Study design

At the beginning of the study, we committed to conducting it in full compliance with the Consolidated Standards of Reporting Trials to ensure transparency, accuracy, and reproducibility. The study was a single-blinded waitlist randomized controlled trial aimed to demonstrate the effectiveness of Mindfulness-Based Relapse Prevention (MBRP) for methamphetamine-dependent young female Chinese adults. The participants were randomly assigned to either the treatment or waiting list group. Those in the treatment group (MBRP) received treatment immediately after the baseline assessment, while those in the control group (waiting list) received treatment after a waiting period of 4 weeks. The study was carried out at a compulsory drug rehabilitation center, in Sichuan Province, China. Chengdu Medical College approved the study (approval number: 2023NO.60). The protocol registration number is ChiCTR2400088849. All the participants voluntarily took part in this study and signed the informed consent forms.

### 2.2 Participants

The target sample size of 60 was determined regarding reduction in drug cravings and increase in trait mindfulness as the primary endpoint using G*Power statistical software. The parameters set for the power analysis were α= 0.05 (two-tailed), 1 - β= 0.95 (power of 95%), and an effect size of 0.15 according to *a priori* estimates for repeated ANOVA. Based on these inputs, the calculated minimum sample size required to detect a significant difference was twenty-two participants per group. Considering a 5% dropout rate, 60 participants were recruited from a compulsory drug rehabilitation center in Sichuan province, China from April to June 2023. The inclusion criteria were follows:

(1) mainly use amphetamine-type substances.
(2) meet the ICD-10 diagnostic criteria for amphetamine dependence.
(3) in good health, with normal vision, normal hearing, and no serious psychiatric illness or brain injury.
(4) aged 18–35 years.
(5) passed through the physiological detoxification period and not on substitution medication, such as methadone.
(6) able to understand the study content completely and participate voluntarily.

Exclusion criteria were:

(1) previous or current serious mental illness (e.g., schizophrenia, bipolar disorder, acute psychosis, etc.).
(2) previous or current serious physical or organic brain disease that may affect cognitive function (e.g., stroke, epilepsy, etc.).
(3) a history of other drug abuse.

### 2.3 Procedure

#### 2.3.1 Experimental procedure

For this randomized controlled trial, we used a web-based random number sequencer (http://www.randomizer.org) to implement randomization. After completing the recruitment, two MA-dependent young adults who missed six treatment sessions for scheduling conflicts were excluded in the analysis. The eligible participants were randomly divided into MBRP and control groups (n = 29 per group). Before the start of the experiment, the subjects were informed in detail of the purpose and process of the experiment, and it was clearly emphasized that they could choose to discontinue the experiment at any time and that participation was voluntary. As well as the center’s usual treatment, the MBRP group will receive MBRP training. To ensure sufficient space for participants and an adequate number of devices, five or six participants constituted one MBRP group, who met twice a week for 4 weeks, for 2 hours each time. In some sessions, MBRP group participants used the mental feedback monitoring balance instrument and wore the head-mounted sensor during the training process, to collect relaxation and concentration data during the training process and determine their trait mindfulness. The content and themes of the training are shown in **Table 1**. The control group only received the center’s usual treatment. Both groups were assessed on all scales, at baseline and immediately after the 4-week program.

**Table 1:** The main content of each intervention session.

Session	Theme	Main practice
Session1	Automatic Pilot and Mindful Awareness	Body scan, Mindfully eat raisins
Session2	A New Relationship with Discomfort	Body Scan, Urge Surfing, Mountain Meditation
Session3	From Reacting to Responding	Awareness of hearing, Breath Meditation, SOBER Space
Session4	Mindfulness in Challenging Situations	Awareness of Seeing, Sitting Meditation: Sound, Breath, Sensation, Thought SOBER Space in a Challenging Situation, Walking Meditation
Session5	Acceptance and Skillful Action	Sitting Meditation: Sound, Breath, Sensation, Thought, Emotion, SOBER Space
Session6	Seeing Thoughts as Thoughts	Sitting Meditation: Thoughts, SOBER Space
Session7	Supporting and Sustaining Well-Being	Kindness Meditation
Session8	Social Support and Continuing Practice	Body Scan, Breath Meditation

#### 2.3.2 Interventions

The MBRP training was supervised by one experienced therapist and two professionally trained psychology graduate students and it was implemented according to the work of Bowen et al. (*Mindfulness-based relapse prevention for addictive behaviors: a clinician guide*). The detailed operationalization of the MBRP is shown in **Table 1**.

### 2.4 Instruments

#### 2.4.1 General information questionnaire

A general questionnaire was designed to collect demographic information about the participants (e.g., age, level of education, marital status, number of drug detoxification attempts, and whether they received drug education prior to admission).

#### 2.4.2 Five-Factor Mindfulness Questionnaire (FFMQ)

The FFMQ was used to evaluate the trait mindfulness of the study participants. Baer et al. developed the scale (15), and Deng translated it into Chinese (16). It has a total of 39 questions, 19 of which are reverse-scored. The revised scale consists of five factors, namely observation, description, action with awareness, acceptance without judgment, and non-reaction to experience. Individuals who score higher on the five factors are more likely to have a higher level of trait mindfulness. The factors of the Chinese version of the FFMQ have good Cronbach’s ɑ values of 0.746, 0.843, 0.794, 0.659, and 0.448, respectively.

#### 2.4.3 Obsessive Compulsive Drug Use Scale (OCDUS)

The OCDUS evaluates psychological craving over a week. The Chinese version of the OCDUS was compiled by Wei Weiquan et al. from the Alcohol Compulsive Drug Use Questionnaire of Franken et al., after deleting some of the questions as appropriate (17, 18). It consists of 13 items on drug use, each of which is scored on a scale of 1–5 scale (items 6 and 13 are reverse-scored), the higher the score, the greater the craving. The Cronbach’s ɑ value of OCDUS is 0.84.

#### 2.4.4 Instruments for measuring physiological indicators

During the training process, the mental feedback monitoring balance instrument captures data on relaxation and concentration, which are physiological indicators that can reflect an individual’s level of mindfulness. Concentration represents the degree of concentration during the practice of mindfulness, which is in line with the concept of “awareness” in MBRP; relaxation reflects the degree of physical and mental relaxation of the user, which is in line with the concept of “acceptance and openness” emphasized in MBRP. The mental feedback monitoring balance instrument can collect real-time concentration and relaxation data during mindfulness exercises through a head-mounted sensor (MindXP™, BJ, CHN). Scores ranging from 0–100 are obtained, with higher scores reflecting greater concentration and relaxation. During the data acquisition process, it is only necessary to contact the arm with the prefrontal Fpl electrode site and to clip the reference electrode to the earlobe for the acquisition of EEG signals to generate real-time relaxation and concentration.

### 2.5 Data analysis

Collected data was organized using Excel, and demographic data of MBRP and control groups were analyzed using SPSS 21.0 (IBM Corp., Armonk, NY, USA) through independent t-test or chi-square test. For the primary outcome, the repeated measures ANOVA with the between-subject factor group and the within-subject factor time were performed. Relaxation and concentration data were collected by the mental feedback monitoring balance instrument. We also examined the differences between the MBRP and control groups in terms of the OCDUS and FFMQ scores at baseline and post-test. The trait mindfulness and craving of MA-dependent female young adults were compared before and after the mindfulness intervention. Differences were considered statistically significant at *P* < 0. 05.

## 3 Results

### 3.1 Demographic data

**Figure 1:**
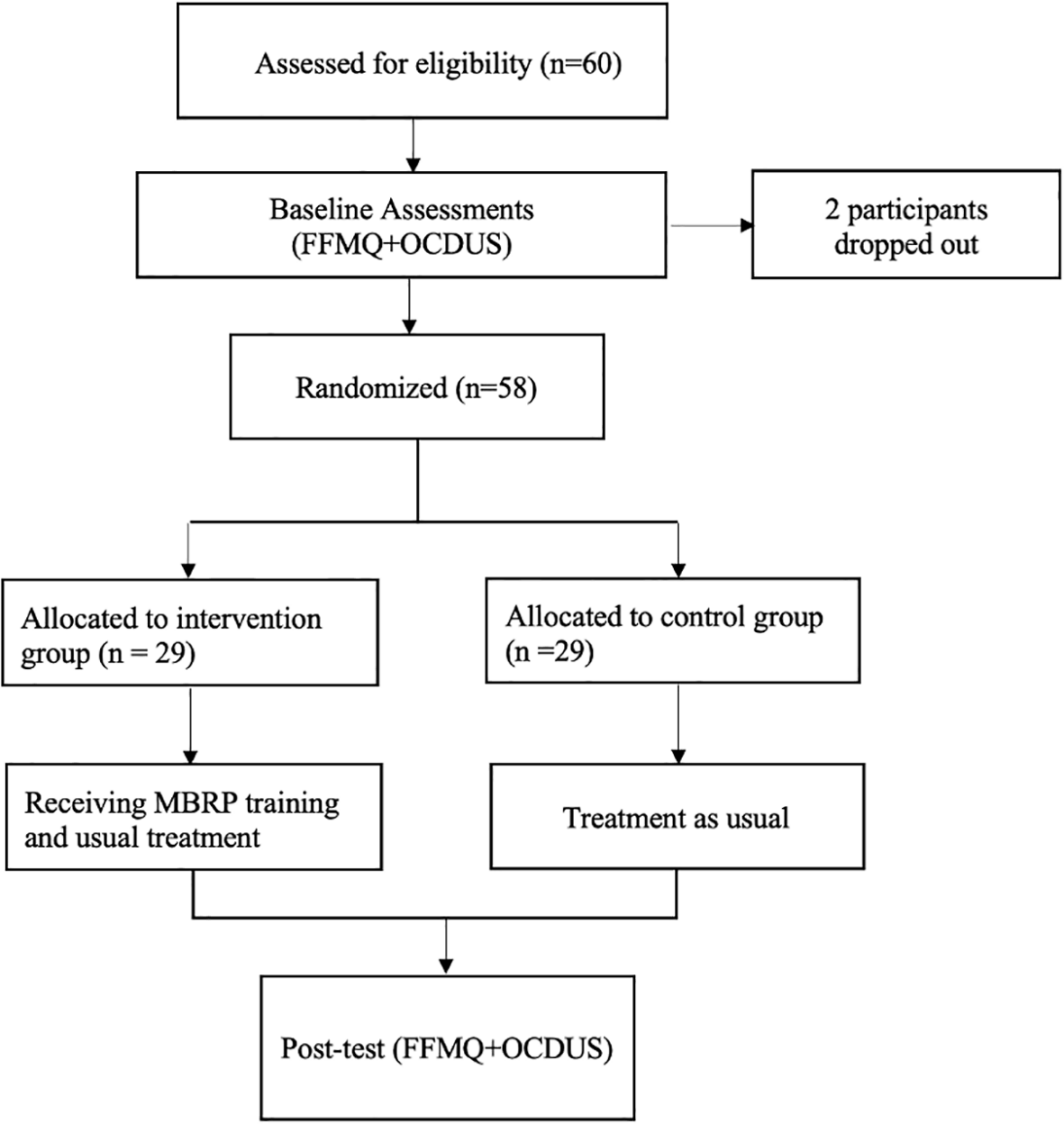
Flow chart of this study.

Sixty MA-dependent female young adults in rehabilitation after physiological detoxification, and who met the enrollment criteria, were recruited. The enrollment process is shown in **Figure 1**. Due to scheduling conflicts during the study, 2 participants dropped out, so 58 MA-dependent female young adults were divided into the MBRP and control groups. There was no significant difference between the two groups in demographic factors. The 29 MBRP group participants had an age of 29.2 ± 4.3 years and the following education levels: 2 (6.9%) cases in elementary school, 15 (51.7) in middle school, and 12 (41.4) in high school and above. Marital status was as follows: single or divorced, 13 (44.8) cases; married, 8 (27.6) cases; divorced, 8 (27.7) cases. The numbers of compulsory isolations were as follows: once, 23 (79.3) cases; more than once, 6 (20.7) cases. Seventeen (58.6) cases had received drug education before admission and 12 (41.4) had not. The 29 control group participants had an age of 29.8 ± 3.5 years, and the following education levels: elementary school, 3 (10.3) cases; middle school, 14 (48.2) cases; high school and above, 12 (41.5) cases. Marital status was as follows: single or divorced, 11 (37,9) cases; married, 11 (37.9) cases; divorced, 7 (24.2) cases. The numbers of compulsory isolations were as follows: once, 25 (86.2) cases; more than once, 4 (48.3) cases. Fourteen (48.3) cases had received drug education before admission and 15 (51.7) cases had not. The differences between the MBRP and control groups in baseline total and dimension scores were not statistically significant (all *P* > 0. 05).

### 3.2 FFMQ score

The differences between the MBRP and control groups in baseline total and dimension scores were not statistically significant (all *P* > 0.05, **Table 2**). After training, the results of repeated measured ANOVAs showed that: (1) the main effect of time on observing, non-judging, and non-reacting were significant [*F*_(1,56)_ = 8.723, *P* = 0.005, *η^2^p* = 0.135; *F*_(1,56)_ = 5.751, *P* = 0.020, *η^2^p* = 0.093; *F*_(1,56)_ = 4.692, *P* = 0.035, *η^2^p* = 0.077]; The further pairwise comparison showed that observing, non-judging and non-reacting scores at the post-test were significantly higher than the baseline(*P* = 0.005, *P* = 0.020, *P* = 0.035). (2) the group × time interaction effect of FFMQ total score was significant [*F*_(1,56)_ = 5.959, *P* = 0.018, *η^2^p* = 0.096]. The *post hoc* analysis of estimated marginal means (EMMEANS) showed that the FFMQ total score differed significantly between the control and MBRP group in the posttest [*F*_(1,56)_ = 4.562, *P* = 0.037, *η^2^p* = 0.075], the results are shown in **Table 3**.

**Table 2:** Baseline comparison of sociodemographic characteristics and all outcome variables between the MBRP group and the control group at baseline.

Variables		MBRP group	Control group	Chi-square/*t*	*P*
Age (years)		29.2 ± 4.3	29.8 ± 3.5	-0.504	0.617
Education (%)	Primary and below	2(6.9)	3(10.3)	0.234	0.889
	Junior school	15(51.7)	14(48.2)		
	High school or above	12(41.4)	12(41.5)		
Marriage (%)	Single or divorced	13(44.8)	11(37,9)	0.707	0.702
	Married	8(27.6)	11(37.9)		
	Divorced	8(27.7)	7(24.2)		
Compulsory isolation times	Once	23(79.3)	25(86.2)	0.483	0.487
	More than once	6(20.7)	4(13.8)		
Whether to receive anti-drug education before isolation	yes	17(58.6)	14(48.3)	0.624	0.420
	no	12(41.4)	15(51.7)		
FFMQ total score		117.45 ± 16.84	117.10 ± 7.75	4.004	0.921
Observing		22.28 ± 7.63	22.28 ± 7.63	3.689	0.334
Describing		20.59 ± 5.38	20.59 ± 5.38	1.100	0.954
Actaware		23.34 ± 5.33	23.34 ± 5.33	0.563	0.417
Non-judging		20.59 ± 5.38	20.59 ± 5.38	1.584	0.942
Non-reacting		27.83 ± 7.47	27.83 ± 7.47	4.305	0.909
OCDUS		33.41 ± 8.17	33.07 ± 6.30	-0.180	0.858

FFMQ, five-factor mindfulness questionnaire, OCDUS, obsessive compulsive drug use scale. *P < 0.05, **P < 0.01.

**Table 3:** The results of the repeated measured ANOVAs MBRP group in all outcome variables.

Variables	Time	MBRP group ± *s*	Control group ± *s*	Effect	*F*	*p*	*η2*
Observing	Baseline	22.28 ± 7.63	20.59 ± 5.38	G	3.318	0.074	0.056
	Immediately post-test	25.66 ± 6.60	21.93 ± 5.87	T	8.723**	0.005	0.135
				G x T	1.618	0.209	0.028
Describing	Baseline	23.34 ± 5.33	23.28 ± 3.52	G	0.856	0.359	0.015
	Immediately post-test	24.90 ± 4.76	22.97 ± 4.31	T	1.554	0.218	0.027
				G x T	3.496	0.067	0.059
Actaware	Baseline	27.83 ± 7.47	29.21 ± 5.19	G	0.640	0.427	0.011
	Immediately post-test	26.93 ± 7.83	28.21 ± 6.17	T	2.320	0.133	0.040
				G x T	0.007	0.935	0.000
Non-judging	Baseline	25.10 ± 6.38	25.00 ± 4.18	G	0.025	0.874	0.000
	Immediately post-test	23.48 ± 5.57	23.21 ± 4.74	T	5.751*	0.020	0.093
				G x T	0.015	0.903	0.000
Non-reacting	Baseline	18.90 ± 5.27	19.03 ± 3.70	G	1.512	0.224	0.026
	Immediately post-test	21.79 ± 5.31	19.28 ± 3.88	T	4.692*	0.035	0.077
				G x T	3.359	0.072	0.057
FFMQ Total score	Baseline	117.45 ± 16.84	117.10 ± 7.75	G	1.471	0.230	0.026
	Immediately post-test	122.76 ± 15.36	115.59 ± 9.55	T	1.833	0.181	0.032
				G x T	5.959*	0.018	0.096
OCDUS	Baseline	33.41 ± 8.17	33.07 ± 6.30	G	1.978	0.164	0.035
	Immediately post-test	29.83 ± 6.94	34.65 ± 6.29	T	1.222	0.274	0.021
				G x T	8.173**	0.006	0.127

FFMQ, five-factor mindfulness questionnaire, OCDUS, obsessive compulsive drug use scale. *P < 0.05, **P < 0.01.

### 3.3 OCDUS score

The MA-dependent female young adults in the MBRP group had a significantly lower posttest (after 4 weeks of training) OCDUS total score (29.83 ± 6.94) compared with that at baseline (33.41 ± 8.17) (*P* < 0.01), whereas the control group’s posttest score (34.65 ± 6.29) was not statistically significantly different from that at baseline (33.07 ± 6.30) (*P* > 0. 05). The difference in psychological craving between the MBRP group (33.41 ± 8.17) and control group (33.07 ± 6.30) was not statistically significantly different from that at pre-intervention (*P* > 0.05; **Table 1**). The results of repeated measured ANOVAs showed that the group × time interaction effect of OCDUS was significant [*F*_(1,56)_ = 8.173, *P* = 0.006, *η^2^p* = 0.127). The *post hoc* analysis of estimated marginal means (EMMEANS) showed that the OCDUS score differed significantly between the control and MBRP group in the posttest [*F*_(1,56)_ = 7.709, *P* = 0.007, *η^2^p* = 0.121], the results are shown in **Table 3**.

### 3.4 Body scan and SOBER exercise scores

Comparing the MBRP group’s body scan scores between sessions 1 and 8, the post-training concentration score (52.10 ± 10.82) was significantly higher than the pre-test score (45.17 ± 9.96) (*P* < 0.01). Comparing the MBRP group’s SOBER scores between sessions 3 and 8, the post-test concentration score (54.79 ± 7.97) was significantly higher than the pre-test score (50.38 ± 5.84) (*P* < 0.05). The post-test relaxation score (60.83 ± 7.21) was significantly higher than the pre-test score (55.03 ± 6.13) (*P <*0.05; **Table 4**)

**Table 4:** Differences between pre-and post-test body scan and breath meditation scores of the MBRP group.

	Body scan				Breath meditation		
Variables	Time	± *s*	*t*	*P*	± s	*t*	*P*
Concentration	Baseline	45.17 ± 9.96			50.38 ± 5.84		
	Post-test	52.10 ± 10.82	-3.050	0.005**	54.79 ± 7.97	-2.515	0.018*
Relaxation	Baseline	55.83 ± 7.92			55.03 ± 6.13		
	Post-test	58.52 ± 6.19	-1.604	0.120	60.83 ± 7.21	-4.439	0.000***

*P < 0.05, **P < 0.01, ***P < 0.001

## 4 Discussion

This study explored the feasibility of MBRP training to reduce psychological craving in MA-dependent female youths. After 4 weeks, the addicts gradually increased their trait mindfulness and became aware of the reasons for their addiction and what methods were effective when craving reappeared. This study is the first to implement MBRP in young, MA-dependent Chinese women, utilizing biological markers to measure mindfulness.

The main effects of time on observing, non-judging, and non-reacting are significant, which indicates that the mindfulness ability of young female MA-dependent adults increased over time. This finding is consistent with a previous study by Brewer et al., which also reported significant increases in FFMQ scores over time for treatment completers in the mindfulness training group based on MBRP (19). The two dimensions of describing, “actaware” did not show significant changes, probably due to the fact that MA-dependent female youths generally received a limited education and have been in a state of emotional repression for a long period (20, 21). Having a higher level of education is associated with a better ability to express and describe emotions (22). On the other hand, drug addicts tend to struggle with emotional expression and have an imbalance in emotional regulation, which can have a negative impact on their ability to describe their feelings. Additionally, studies have shown that unemployed individuals tend to score lower on the action awareness scale compared to those who are employed. Interestingly, a significant number of drug addicts in this study were found to be unemployed (23). Thus, a longer period may be needed for improvement in these aspects. It is also important to note that the interaction between time and group had a significant impact on both mindfulness ability and psychological cravings. These findings indicated that MBRP training had shown significant improvement in the trait mindfulness of young female MA-dependent adults, while also reducing their cravings over time than the control group. MBRP training encourages the participants to focus on the present moment and pay attention to the associated thoughts and feelings with acceptance, openness, and a non-judgmental attitude, and to pay more attention to their body, emotions, and thoughts in high-risk situations, thus attenuating the “automated” response between emotion and drug use and reducing psychological cravings and the likelihood of relapse, in line with the results of previous studies (24, 25).

Concentration and relaxation, as indexed by the mental feedback monitoring balance instrument, can serve as a measure of mindfulness. Past studies have mostly used self-report scales to assess individual trait mindfulness changes, such as the FFMQ and MMAS. To improve the objectivity of the present study, the mental feedback monitoring balance instrument can be a more detailed assessment of mindfulness levels and clarify the relationship between mindfulness and changes in craving more clearly and enhance ecological validity. Brain–computer interface technology has been widely used in the field of mindfulness in recent years, and measurement of biological parameters using wearable devices can help the public better understand the effects of mindfulness practice on the body. The acquisition process only requires contacting the support arm for EEG acquisition with the prefrontal Fpl of the brain and clamping a reference electrode to the earlobe to acquire EEG signals, making it simple to measure a wide variety of brainwave data (29). To confirm the effectiveness of the MBRP training, we utilized the mental feedback monitoring balance instrument to compare body scanning exercise data between sessions 1 and 8, as well as the breath meditation exercise data between sessions 3 and 8. The findings indicate that after prolonged MBRP training, whether in the form of body scanning or breath meditation exercises, the MBRP group experienced a notable boost in concentration. Although relaxation also increased during the body scanning exercise, the change was not statistically significant. This could be since most participants reported lying down to be a more relaxing position during the body scan exercise in session 1 compared to the sitting or standing position in the SOBER exercise, creating a potential ceiling effect. Our research provides valuable insights into the effectiveness of mindfulness-based relapse prevention (MBRP) training for young female adults who are dependent on methamphetamine. However, it is important to acknowledge the limitations of our study. Our research utilized a 4-week training program (26, 27), and there is ongoing debate regarding the optimal frequency of mindfulness training (28). A further study should be conducted with a larger sample size to explore the impact of a more intense schedule. It is worth noting that consistent and ongoing mindfulness practice is essential for achieving optimal results. While mindfulness prevention training can lead to positive changes during compulsory drug treatment, individuals may face new challenges and stressors upon returning to society. Therefore, it is crucial to investigate the long-term effects of mindfulness training on addiction recovery and to explore how individuals maintain and enhance their mindfulness practice post-treatment. Additionally, it is important to assess whether mindfulness practice assists individuals in managing cravings when faced with real-life challenges.

In conclusion, the present study implemented an MBRP intervention for female MA-dependent young adults. In addition to a traditional self-report scale, an instrumental measure was introduced to assess mindfulness ability. The results confirmed that the MBRP training effectively reduced drug craving in MA-dependent young adults. Additionally, it facilitated trait mindfulness and non-judgmental acceptance of negative thoughts and emotions, while reducing negative cognitive and affective patterns. This led to improved physical and mental health, and a significant reduction in impulsive substance abuse. However, since mindfulness training requires long-term, persistent practice, and drug addicts face many challenges in life after discharge, its long-term effects need further research.

## Data Availability

The original contributions presented in the study are included in the article/supplementary material. Further inquiries can be directed to the corresponding author.
